# A framework for personal protective equipment use in laboratories: regulatory compliance and employee protection

**DOI:** 10.3389/fpubh.2025.1586491

**Published:** 2025-07-17

**Authors:** Segaran P. Pillai, Sarah Bradberry, Marisa Newcomer, Tanya Pittas, Kelsey Mathern

**Affiliations:** Office of Occupational Safety and Health, U.S. Food and Drug Administration, U.S. Department of Health and Human Services, Washington, DC, United States

**Keywords:** personal protective equipment, laboratory safety, respiratory protection, hand protection, face protection, hearing protection, body protection, foot protection

## Abstract

The Occupational Safety and Health Administration (OSHA) Personal Protective Equipment (PPE) Standard, outlined in 29 CFR 1910 Subpart I states that Protective gear - including personal protective clothing, respiratory equipment, and barriers or shields- must be supplied, properly used, and kept clean and functional whenever there are hazards present. These hazards may arise from processes, environmental conditions, chemicals, radiation, or mechanical sources that could potentially cause injury or harm bodily functions through skin contact, inhalation, or absorption. Employers must supply PPE to their employees as determined by hazard assessments of the workplace and duties performed by employees. Employees for their part are required to use PPE as instructed to reduce exposure to occupational hazards and the likelihood of injury or illness, as appropriate. PPE includes all clothing (e.g., coats, gowns, gloves, shoe covers, boots) and other work accessories (e.g., respirators, face shields, safety glasses, goggles) intended to act as a barrier against workplace hazards or to be worn for protection, serving as the last line of defense in the hierarchy of safety controls. The OSHA PPE Standard includes requirements for selection of equipment, training requirements, records management, PPE selection guidelines and potential risk and hazard assessment. OSHA has standards that require such equipment meet or be equivalent to standards developed by the National Institute for Occupational Safety and Health (NIOSH) or the American National Standards Institute (ANSI). This article provides an overview of the types of PPE, and considerations for their selection and use to address laboratory hazards.

## Introduction

Hazard control and risk mitigation is typically maintained through five primary control measures: (1) Elimination – removal of the hazard; (2) Substitution – replacement of the hazard; (3) Engineering Controls – isolating people from the hazard (ex. biological safety cabinet, laboratory ventilation hood); (4) Administrative controls – changing the way people work; and (5) Personal Protective Equipment (PPE) (1). Primary barriers, also known as primary containment, are methods and equipment designed to prevent the release of biological material. They create a physical barrier between the hazardous material and the worker or the environment. Primary barriers range from a biological safety cabinet (BSC) to PPE such as a lab coat. When substitution or elimination of potential hazards is not possible, and engineering controls are insufficient and administrative controls do not offer sufficient protection thorough assessment, PPE serves as a secondary means of protection. PPE should not be relied on as the sole method of protection, but rather should be used in conjunction with substitution of hazards, engineering controls, administrative controls which should include best practices. Thus, PPE should be used as a secondary measure to protect laboratory personnel and other workers from injury or exposure to hazardous materials. Employers must supply PPE to their employees to ensure adequate protection and to mitigate potential exposures and hazards. PPE includes all clothing (e.g., coats, gowns, gloves, shoe covers, boots) and other work accessories (e.g., respirators, face shields, safety glasses, goggles) designed to serve or be worn as a barrier against workplace hazards. Workers who may require PPE include research and clinical laboratory workers, maintenance and housekeeping personnel, and healthcare workers.

The OSHA PPE Standard mandates that the following be considered when implementing a PPE program: (1) Hazard assessment and equipment selection, (2) Employee training, (3) Recordkeeping requirements, (4) Guidelines for selecting PPE, and (5) Certification of hazard assessment (2).

The guidelines presented are based on U.S. OSHA standards. These are not international laws, and other countries are not legally required to follow them. However, they may choose to adopt or adapt these guidelines within their own regulations.

## Hazard assessment and equipment selection

PPE selection starts with a thorough hazard assessment by the employer to identify existing or potential hazards that may necessitate the use of PPE. Following the assessment, a review of the appropriate regulations, guidelines, and standards related to appropriate PPE would be prudent. OSHA has issued several standards that address PPE requirements, including employee training, selection factors, and record keeping. Table 1 captures the OSHA standards that address PPE requirements inclusive of what would be pertinent for the laboratory environment (3). Along with OSHA standards, the Biosafety in Microbiological and Biomedical Laboratories (BMBL) outlines the recommended PPE for laboratories ranging from Biosafety Level (BSL) 1 through BSL-4 (3). The Association of Public Health Laboratories decisions on PPE can also serve as a useful guide for implementing a good PPE program (4).

**Table 1:** OSHA standards for PPE in laboratory environment.

Title	Standard
General Industry: PPE Requirements	29 CFR 1910.132 (2), 29 CFR 1910.133 (9), 29 CFR 1910.134 (6), 29 CFR 1910.135 (23), 29 CFR 1910.136 (14), 29 CFR 1910.137 (15), 1910.138 (16)
General PPE Requirements	29 CFR 1910.132 (2)
Protection Standard: Eye and Face	29 CFR 1910.133 (9)
Protection Standard: Respiratory	29 CFR 1910.134 (6)
Protection Standard: Foot	29 CFR 1910.136 (14)
Protection Standard: Electrical	29 CFR 1910.137 (15)
Protection Standard: Hand	29 CFR 1910.138 (16)
Standard for Bloodborne Pathogen	29 CFR 1910.1030 (24)
Laboratory Standard	29 CFR 1910.1450 (25)

The OSHA standards require employers to provide the appropriate PPE at no cost to employees (in most cases) and ensure that employees use the proper PPE when there is a risk of exposure, illness, or injury (2). These standards also detail specific provisions for various types of PPE. Equipment, such as respirators, must be properly fitted, kept in clean and serviceable condition, and periodically inspected to assess the effectiveness of the PPE (5).

When evaluating and selecting the most appropriate PPE for a specific procedure or environment, several factors should be taken into consideration which are captured in Table 2.

**Table 2:** Factors to consider when selecting the appropriate PPE.

PPE selection factors	The type of hazardous materials being used (e.g., biological, chemical, radiological, laser, nanomaterials etc.) along with their concentration and quantity.
	Risk associated with the materials.
	The potential routes of exposure.
	The type of PPE that offers optimal protection.
	The permeation and degradation rates of PPE against such hazardous materials.
	The length of time for the effective use of the PPE.
	The comfort and fit of PPE to ensure optimal protection.

Principal Investigators and laboratory supervisors should assess the hazards associated with all job tasks under their supervision and develop appropriate policies and procedures, including minimum PPE requirements for personnel working in or entering their laboratories. All personal protective equipment should be properly stored, maintained, sanitized when necessary, and inspected or replaced if damaged or expired. It is highly recommended that PPE meet NIOSH (National Institute of Occupational Safety and Health) and ANSI standards to ensure adequate quality of protection and efficacy standards.

## Training

It is essential that employees are trained in the proper selection, use, and maintenance of PPE, as well as understanding its limitations before use. This training can be delivered through various methods such as videos, web-based presentations, group sessions, and handouts. Periodic refresher training should be provided to employees, contractors or visitors as required by regulations or more frequently if needed.

Training should cover the following key topics: when PPE is necessary, which PPE is required, how to correctly don (put on), doff (remove), make adjustments as needed, PPE limitations, proper cleaning techniques, care, maintenance, effective lifespan, and disposal of the equipment. Additionally, the specific PPE required for tasks involving hazardous biological, chemical, radiological, laser, nanomaterials, and physical hazards must be outlined in laboratory standard operating procedures, and employees must be trained accordingly. Before employees are permitted to perform work using assigned PPE, employee must show a clear understanding of their PPE training (2).

It is required to document and maintain records of all PPE training, including details of the information covered, the names of the individuals trained, the type of training provided, and the training dates (6).

When conducting risk assessments for PPE, it is important to consider the diverse range of hazards and PPE types available to mitigate those hazards.

## Hearing protection

The OSHA Occupational Noise Exposure Standard 29 CFR 1910 Subpart G (7) requires a hearing conservation program when employee noise exposures reach or exceed an 8-h time-weighted average sound level of 85 dBA (decibels, A-weighted scale).

Noise (defined as unwanted sound) can be characterized as continuous, intermittent, or impulse. Continuous noises are often found in equipment areas related to laboratory operations where the equipment runs constantly and can sometimes be outfitted with engineering controls to reduce noise levels. Intermittent noises are most associated with portions of laboratory process that use equipment such as grinders or vivarium areas that experience intermittent animal noise. Impulse noises begin almost instantly and are short in duration. Impulse noises include sounds such as grinding of material for sample processing and sonication. Employees exposed to hazardous noise levels in the workplace are at risk of developing noise-induced hearing loss. While this condition is preventable, once it occurs, it is permanent. There are several types of hearing protection devices available, including (1) Earplugs: These are inserted into the ear canal to block out noise. They can be disposable or reusable and may be made of foam, silicone, or other materials; (2) Earmuffs: These are worn over the ears and are held in place with a headband. They provide a physical barrier to block out noise; and (3) Noise-canceling headphones: These are worn over the ears and use electronic signals to cancel out background noise. They are commonly used in industries such as aviation or construction.

Prior to using these devices, it is important to implement engineering and/or administrative controls to reduce noise levels in the workplace. This may involve installing sound-absorbing materials or using quieter machinery. Employers are responsible for providing appropriate hearing protection based on factors including comfort, type, size, and their tested ability to attenuate actual noise levels, and training employees on their proper use. It is also important to properly maintain and store hearing protection, and to replace it when it becomes damaged or no longer fits properly.

## Protective clothing

Protective clothing includes lab coats and other garments such as aprons to protect the frontal section of your body, boots to protect the feet, shoe covers to prevent the transfer of hazardous materials, liquid-resistant coveralls to prevent liquid penetration, and similar items. These items help protect street clothing from contamination and splashes of biological, chemical, radiological, and nanomaterials, while also offering additional protection against physical hazards such as lasers and extreme temperatures. Principal Investigators and laboratory supervisors should enforce appropriate procedures in the laboratory such as prohibiting the wearing of short length garments or open-toed shoes by laboratory personnel, visitors, or for individuals working in or entering laboratories under their supervision to protect against various physical hazards associated with laboratory work. These hazards include broken glass, dropped laboratory chemicals, and splashes.

OSHAs General Requirements for Personal Protective Equipment Standard 29 CFR 1910.132 (2) requires protective clothing when necessary to protect employees from task-specific hazards, including chemical, biological, or radiological hazards, or mechanical irritants, or the environment itself.

When selecting protective clothing, it’s crucial to consider the specific hazards and the level of protection needed, the material’s resistance and provide protection against those hazards, the comfort of the clothing, whether it is disposable or reusable, and how easily it can be removed in an emergency. Table 3 notes standards to ensure protective clothing meets the requirements of common laboratory hazards (8).

**Table 3:** Examples of applicable hazards and relevant standards.

Examples of applicable hazards	Relevant standards	Standard measurements
Non-hazardous materials	Non-applicable	Non-applicable
Small amounts of potentially hazardous aqueous solutions or infectious materials	AATCC Method 42	Resistance to water penetration by impact
Human or animal blood and body fluids	ASTM F1670	Penetration by synthetic blood
Bloodborne pathogens including: Hepatitis B Virus Hepatitis C Virus Human Immunodeficiency Virus	ASTM F1671	Bloodborne pathogen exposure Note: Standard is more rigorous to pass than ASTM F1670
Chemicals are tested individually	ASTM F903	Liquid chemical barrier
Flammable, pyrophoric liquids or solids	NFPA 701 or 2,112 Flame Propagation Tests	Flammable

Personnel should seek guidance from an occupational safety and health officer when planning their experiment that may require specialized protective clothing or when they have questions about the most suitable protective gear to use. Always confirm manufacturer specifications of protective clothing meets standards for hazards identified during the risk assessment.

It is prudent to wear long, fitted garments to prevent skin exposure and sturdy shoes in all laboratories and mechanical areas to avoid exposure or injury from physical hazards. Shorts, garments with excessively loose fabric, and open-toed shoes should be prohibited in laboratories.

## Eye and face protection

Eye protection is among the most crucial and critical PPE one should consider. The OSHA Eye and Face Protection Standard 29 CFR 1910.133 (9) mandates the provision for eye and face protection that complies with specific standards. When selecting eye protection and prescription eyewear, it is essential to verify that they comply with American National Standards Institute (ANSI) Z87.1–2003 (10), ‘American National Standard Practice for Occupational and Educational Eye and Face Protection,’ and are clearly marked with the manufacturer’s name (11).

Eye protection is a crucial aspect of personal protective equipment (PPE) and should be used to safeguard employees, contractors, and visitors from various hazards, including splashes, flying particles from shattered glass, acids, or caustic liquids, chemical, gases or vapors, biological materials, radiological materials, nanomaterials, and harmful exposure to ultraviolet light or non-ionizing radiation such as lasers. Eye protection should be worn at all times where chemicals, biologicals, radiological, lasers and nanomaterials are being handled or stored, even if not directly working with such materials, and when these hazards are present. Principal Investigators and laboratory supervisors should make, use of eye protection a mandatory requirement for all laboratory personnel, including contractors and visitors, working in or entering laboratories under their control.

### Prescription safety eyewear

OSHA regulations mandate that employees who wear prescription glasses while working with hazards materials must use eye protection that includes their prescription in its design or wear eye protection that fits over their prescription glasses (such as goggles or face shields) without affecting the proper alignment of the prescription lenses or the protective lenses. All prescription eyewear must meet the requirements of ANSI Z87.1-2020 (12).

Safety glasses offer protection against moderate impact and particles, making them suitable for tasks like scaling, grinding, sawing, minor splashes, and handling broken glass of hazardous and flying objects or materials. For individuals who require corrective lenses, prescription safety glasses should be made available. However, safety glasses are not adequate for processes such as stirring, pouring, or mixing, where splashes of hazardous materials may occur. In these situations, splash goggles should be used instead.

Splash goggles provide protection against chemical, biological, radiological or nanomaterials splash hazards, concentrated corrosive materials, and bulk transfer of hazardous materials. Goggles come with clear or tinted lenses, fog-resistant coatings, and options for vented or non-vented frames.

Welder’s and Chipper’s Goggles offer protection against harmful light rays, sparks, scaling, and metal splashes. They feature impact-resistant lenses and are available in graduated shades. Chipper’s/grinder’s goggles protect against flying particles and feature dual protective eyecups with impact-resistant clear lenses and separate cover plates.

Face shields are designed to provide face protection. When used with safety glasses or splash goggles, face shields provide added protection to the eyes and face. Face shields feature adjustable headgear and can be equipped with a tinted or clear lens, or a mesh wire screen. They are designed to protect the entire face from hazards like chemicals, biological materials, radiological material, and nanomaterials. Additionally, face shields offer protection against physical hazards such as flying particles, splattering liquids, metal sparks, and splashes. Face shields should never be worn on their own and are not a replacement for proper eyewear. They must always be used in combination with a primary form of eye protection.

Laser eye protection is designed to match and protect the eyes from spectral frequency or wavelength of the laser source. As a result, one type of safety glasses is not adequate to protect against all laser outputs. Utilize manufacturer specifications to ensure that the glasses protect within the wavelength of the laser source and fall within the ANSI Standard Z136 for Laser Safety. Laser eye protection is required to be inspected at least once a year for damage to the attenuation material such as pitting, cracking, and discoloration; mechanical integrity of the frame; and light leaks and coating damage (13).

## Respiratory protection

OSHAs Respiratory Protection Standard 29 CFR 1910.134 (6) requires employers to provide respirators to employees when necessary to protect their health and respiratory and inhalation risk. Respirators can provide protection against physical, chemical, nanomaterial and biological hazards present in atmospheres if used correctly and are properly fitted. Prior to initial respirator use, a medical evaluation must be conducted to ensure use of a respirator does not cause a health risk to the user. Use of a tight-fitting respirator also requires a fit test to ensure a proper seal and protection of the user prior to initial use. While fit testing is required annually thereafter, if the respirator required changes, or when an individual’s facial structure changes, medical clearances are to be conducted periodically. Respiratory Protection Training is also required prior to use of a respirator and annually thereafter. Respiratory protection encompasses all NIOSH-approved respirators, including disposable respirators, N-95 masks, half-face and full-face respirators, and powered air-purifying respirators (PAPRs). Respiratory protective devices generally fall into two categories: air-purifying respirators, which filter or absorb contaminants from the breathing air, and air-supplied respirators, which provide clean air from an external source or storage tank. Types of respirators include:

- Filtering facepiece respirators: These are disposable half-face respirators that filter out airborne particles. They do not provide protection from gases and vapors. Filtering facepiece respirators, such as N-95 s, may be used to protect users from animal allergens or potential biological hazards when engineering controls alone are not sufficient.
- Half-face respirators: These respirators cover the nose and mouth and are held in place with straps. They can be fitted with various filters to provide protection against specific hazards.
- Full-face respirators: Full-face respirators provide protection to the eyes, nose, and mouth and should be used when working with hazardous dusts, fogs, or mists. These cover the entire face and are typically used in situations where the eyes may also be at risk of exposure to hazards like chemical, biological, or nanomaterials, spills, or splashes.
- Powered, air-purifying respirators (PAPR): These respirators feature a battery-powered filtration mechanism that draws air through attached filters and chemical-absorbing cartridges or canisters by removing hazardous materials and delivering it to a facepiece. In the case of loose-fitting PAPRs the filtered air is supplied to the hood instead of a facepiece. Different filters are required to protect against different hazards. PAPRs may be used where there is aerosol exposure risk in biological safety laboratory where engineering controls are not sufficient.
- Supplied-air respirators and self-contained breathing apparatus (SCBA) respirators: These types of respirators are utilized in situations where the air may be contaminated, or oxygen levels are low. SCBA’s are typically used by firefighters and other emergency responders.

The employer must use any feasible engineering controls, such as ventilation systems or local exhaust hoods, that remove contaminants, rather than relying on respirators. Appropriate respirators should only be used when effective engineering controls are not accessible or available to provide the necessary protection.

## Foot protection

OSHAs Foot Protection Standard 29 CFR 1910.136 (14) requires employers to provide protective footwear to employees working in areas where there is a risk of foot injuries from falling or rolling objects, objects piercing the sole, or electrical hazards. Wearing proper foot protection is essential to prevent injuries from chemical spills and physical hazards. This includes wearing closed-toed shoes, such as leather or chemically resistant rubber boots where hazardous chemicals or physical hazards are present.

To prevent possible biological, chemical, or radiological contamination, booties may be used to cover street footwear, or dedicated facility shoes may be kept and worn, per a risk assessment and associated SOPs.

Avoid wearing sandals, mules (with exposed heels), or perforated shoes, as they do not offer sufficient foot protection against chemical spills or physical hazards. In some cases, steel-toed or composite-toed shoes may be required when working with heavy equipment or materials. Composite-toed shoes offer similar protection to steel-toed shoes but are lighter and non-metallic.

## Protection against electrical shock

OSHAs Electrical Protective Equipment Standard 29 CFR 1910.137 (15) applies to the proper use of PPE when exposed to electrical hazards that cannot be sufficiently controlled, contained, or managed through engineering controls. Some equipment used when an employee is working on or near live electrical parts include:

- Insulated gloves made of materials that provide insulation against electric shock. Gloves must be voltage-rated and tested per ASTM D120. Rubber insulating gloves are usually worn with leather protectors.
- Insulated sleeves made of insulating material and worn over the arms.
- Insulated blankets made of insulating material and are used to cover electrical equipment or machinery to prevent accidental contact with live parts.
- Insulated mats made of insulating material and are used to cover floors or other surfaces to prevent grounding.
- Hearing protection when arc blast or high decibel noise is possible which may include foam earplugs or earmuffs rated to reduce harmful noise exposure.
- Insulated tools although not worn, but essential should be rated for electrical work.
- Electrical hazard rated footwear to protect from step potential and electrical shock through feet.
- Safety glasses or goggles for eye protection from arc flashes or flying debris and should meet ANSI Z87.1 standard.
- Hard hats to provide impact and electrical insulation.
- PPEs with assigned Category (CAT) 1–4 based on Arc Thermal Performance Value (ATPV). CAT 1 with a minimum ATPV rating of 4 cal/cm^2^ requires a typical PPE of Arc-rated shirt and pants, safety glasses, hearing protection, leather gloves; CAT 2 with a minimum ATPV rating of 8 cal/cm^2^ requires a typical PPE of Arc-rated shirt and pants, safety glasses, hearing protection, leather gloves, arc-rated face shield with balaclava, voltage-rated gloves and hard hat; CAT 3 with a minimum ATPV rating of 25 cal/cm^2^ requires full arc-rated suit, arc flash hood voltage-rated gloves and tools, and full-body coverage; CAT 4 with a minimum ATPV rating of 40 cal/cm^2^ requires heavy-duty arc-rated suit and hood, full PPE for body coverage and high-energy tasks as per NFPA 70 E Table 130.7(C) (15) (c).

## Hand protection

OSHAs Hand Protection Standard 29 CFR 1910.138 (16) requires employers to provide appropriate hand protection and be made available to employees when needed. Most hand-related accident can be classified into four primary hazard categories: exposure to hazardous materials, abrasions, cuts, and heat/cold. Appropriate hand protection should be worn whenever there is a potential hazard from biologicals, chemicals, radiological, nanomaterials, cuts, lacerations, abrasions, punctures, burns, or extreme temperatures.

Hand protection must be worn while using chemicals that can be easily absorbed through the skin or are highly hazardous, including “select carcinogens,” reproductive toxins, radiological materials, nanomaterials, biological materials, and substances with a high degree of acute toxicity or infectivity that can cause accidentally be transferred to mucosal surfaces to cause an infection. No single type of glove offers optimal protection against all hazardous materials or fully prevents degradation and permeation by these substances. Gloves must be replaced when compromised or regularly, considering factors such as the type and concentration of hazardous materials, the gloves’ performance characteristics, the conditions and duration of use, the hazards involved, and the length of time the hazardous material has been in contact with the glove.

All glove materials will eventually be permeated by different hazardous substances. However, they can be used safely for limited periods if the specific use, as well as factors like thickness, permeation rate, and duration of use, are properly understood.

### Hand protection — use of gloves

Gloves should be worn whenever significant hazards such as chemicals biological, radiological exposures, cuts, lacerations, abrasions, punctures, burns, biologicals, or harmful temperature extremes are encountered. Disposable gloves should be discarded after each use. Do not wear gloves outside the hazard area as they can pose a risk for transfer or cross-contamination.

Guidelines for using gloves include wearing appropriate gloves whenever there is potential for contact with hazardous materials, inspecting gloves for any holes, cracks, degradation, or contamination prior to each use. Gloves that are found to be damaged or in questionable condition should be discarded immediately. Additionally, gloves should be replaced periodically based on how often they are used and their permeability to the substances being handled. Reusable rubber gloves should be cleaned after each use following the manufacturer’s instructions. Disposable gloves should be carefully removed and discarded after each use or whenever they become contaminated or compromised. Reusing disposable gloves is not recommended as is increases the risk of cross-contamination, potentially compromising both research and the safety and health of the employee. To avoid chemical, biological, or radiological material contamination, gloves should always be removed before leaving the hazard area and should never be worn outside of the laboratory, including when grabbing a door handle, or using an elevator. If you need to wear a glove to hold something, remove one glove and use the barehand to touch door handles, disengaging locks.

When removing gloves, grasp the cuff of the left glove with the gloved right hand and pull off the left glove. Then, invert the right glove over the left glove, enclosing both in the palm of your hand, and dispose of them. Be sure to wash your hands thoroughly with soap and water after glove removal and before leaving the laboratory.

### Hand protection — selection of gloves

Before handling with hazardous materials, always review the manufacturer’s instructions and warnings on the container labels and Safety Data Sheets (SDSs) for chemicals or understand the biological, radiological, laser or nanomaterial properties, their associated hazards and risk, their mode or route of infection, exposure, or absorption. The SDSs may recommend specific glove types. If the recommended glove type is not indicated on the SDS, consult the s glove selection chart provided by the manufacturer, which usually includes a list of commonly used chemicals tested with the manufacturer’s various glove types. Remember that different manufacturers use different formulations, so always consult with the specific manufacturer of the glove you intend to use. If the manufacturer’s glove chart does not include the specific chemical you will be using, reach out to the manufacturer directly and consult with their technical representatives to identify the best glove for your application. It is important to recognize that not all chemicals or mixtures have been tested by glove manufacturers. In such cases, it is especially crucial to contact the manufacturer directly. In some instances, you may need to consult with a testing laboratory that specializes in identifying the most resistant glove material for the specific chemical you are planning to use it for.

### Hand protection — double gloving

One way to enhance protection while wearing gloves is to use a technique called “double-gloving.” This practice involves wearing two pairs of gloves, one over the other, to offer an extra layer of protection. If the outer glove becomes contaminated, begins to degrade, or tears, the inner glove will continue to provide secondary protection until both gloves are removed and replaced. At the first sign of contamination or degradation, immediately remove and discard the outer glove, then double-glove with a fresh set. If the inner glove shows signs of contamination or degradation, remove both pairs of gloves, wash your hands thoroughly, reevaluate to determine if these are the best gloves to provide the necessary protection. If so, double glove with a new set. This practice is usually used in clinical research laboratories when dealing with infectious biological agents, hazardous chemicals, radiological or nanomaterials.

It is essential to regularly check outer gloves for signs of degradation, such as small perforations, color, or texture changes. If such changes are observed and immediately remove and dispose of the contaminated gloves. One approach to double-gloving is to wear a thin disposable glove beneath a thicker glove, where the outer glove acts as the primary protective barrier, and the inner glove serves as a secondary barrier in case the outer gloved degrades or allows chemicals, biologicals, radiological, nanomaterial or to permeate. Alternatively, you can wear a more durable, thicker glove as the inner layer for better dexterity and a thinner disposable glove as the outer layer but remember to change the outer glove frequently.

When dealing with mixtures of chemical or physical hazards, the first step is to check for chemical compatibility. In some cases, double-gloving with two different types of gloves made from distinct materials may be necessary to protect against the possibility of the outer glove becoming permeated by one of the hazards. The glove material selected should depend on the specific chemicals involved in the mixture, so it’s crucial to refer to the chemical manufacturers’ glove selection charts before choosing the appropriate gloves. Furthermore, when working with highly pathogenic materials, double-gloving is commonly practiced to prevent hand contamination to prevent cross contamination or accidental transfer of material from the hazard to the other surfaces or mucosal surfaces.

### Hand protection — types of gloves

Different types of gloves can be used to protect against various hazards, including chemicals, biological agents, radiological materials, nanomaterials, abrasions, cuts, and heat/cold.

#### Fabric gloves

Fabric gloves, made from materials such as cotton or fabric blends, are not suitable for protection against chemical, biological, radiological or nanomaterial hazards. These gloves are mainly designed to enhance grip when handling slippery objects or to offer insulation against heat or cold. However, the fabric can absorb and retain hazardous materials potentially exposing the user’s hands to the hazard. It is important to use gloves specifically designed for protection against chemicals, biologicals, nanomaterials, or radiological materials when working with such hazardous substances.

#### Leather gloves

Leather gloves are not suitable for handling or protection against biological, chemical radiological or nanomaterial hazards. Although these gloves may offer some protection against injuries from rough surfaces, sparks, or cuts from sharp objects, they do not provide resistance to the absorption of chemicals, biological agents, nanomaterials, or radiological materials. The leather material can absorb and retain hazardous substances, potentially exposing the user’s hands to these risks. It is important to use gloves specifically designed for protection against chemicals, biologicals, nanomaterials, or radiological materials when working with such hazardous substances. Leather gloves may be used with an insulated liner for electrical work, but they should not be considered adequate protection against chemical, biological, nanomaterial, or radiological hazards.

#### Metal mesh gloves

Metal mesh gloves are designed to protect hands from scratches and cuts that may be caused by sharp objects such as knives and cutting tools. They are unsuitable for use when working with biologicals, chemicals, radiological or nanomaterials as the metal mesh will not provide sufficient protection against exposure to such hazards.

#### Cryogenic gloves

Cryogenic gloves are specifically designed to protect hands from extremely low temperatures, such as those encountered when handling dry ice (solid carbon dioxide) or working with liquid nitrogen, argon and helium, other cryogenic substances. Cryogenic gloves are essential for protecting the hands from cold-induced injuries, such as frostbite and cryogenic burns.

#### Chemical resistant gloves

Chemically resistant gloves are made from various materials (see Table 4). When a glove comes into contact with a chemical, it should be removed and replaced as soon as practical. Gloves designed for incidental contact may not provide adequate protection for prolonged exposure to certain chemicals. Always consult the manufacturer’s recommendations or glove selection charts before choosing chemically resistant glove (17–19).

**Table 4:** Guidelines for different glove materials.

Glove material	Resistance
Natural Rubber Latex	Resistant to ketones, alcohols, caustics, and organic acids.
Neoprene	Resistant to mineral acids, organic acids, caustics, alcohols, and petroleum solvents.
Nitrile	Resistant to alcohols, caustics, organic acids, and some ketones. Acceptable as an alternative to latex gloves for handling biological agents.
Norfoil	Rated for chemicals considered highly toxic and chemicals that are easily absorbed through the skin. These gloves are chemically resistant to a wide range of materials that readily degrade other glove materials. These gloves are not recommended for use with Chloroform. Common brand names include: Silver Shield by North Hand Protection, 4H by Safety4, or New Barrier by Ansell Edmont.
Polyvinyl chloride (PVC)	Resistant to mineral acids, caustics, organic acids, and alcohols.
Polyvinyl alcohol (PVA)	Resistant to chlorinated solvents, petroleum solvents, and aromatics.

## Gloves for preventing chemical, biological, radiological or nanomaterial exposure

Latex gloves should only be used for handling non-hazardous chemical, biological, radiological or nanomaterials activities in clean room environments. They are suitable for medical or veterinary applications. Latex gloves may also be used for handling very diluted chemicals or solutions containing less than 1% of hazardous materials or suspected human carcinogens. Latex gloves can induce sensitivity over time leading to severe allergic reactions in some individuals which may include nasal, eye, or sinus irritation, hives, shortness of breath, coughing, wheezing, or unexplained shock (20). Therefore, hypoallergenic, non-powdered gloves should be used whenever possible. Alternatives to latex gloves are listed in Table 3.

The recommended glove material for handling biological agents and nanomaterials is nitrile, however, results of the risk assessment of all materials and procedures used may contraindicate nitrile glove use (21). This is often the case when nitrile gloves do not provide appropriate resistance to chemicals used in biological agent or nanomaterial manipulation (3).

Radiological materials present an additional challenge. To protect against radiation, lead gloves are recommended to reduce occupational exposure through the hands (22).

## Conclusion

Personal Protective Equipment (PPE) should not be the sole means of protection. It should be used alongside with good engineering practices, administrative controls, and good work habits.

The Occupational Safety and Health Administration (OSHA) Personal Protective Equipment standard, outlined in 29 CFR 1910 Subpart I (2), includes requirements for selecting equipment, employee training, records management, guidelines for PPE selection, and hazard assessments.

Personal protective equipment, such as gloves, eye protection, lab coats, face shields, aprons, and boots, must be supplied to employees based on a job hazard analyses to protect against exposure to hazardous agents. PPE should be easily accessible and, in most cases, provided at no cost to the employee.

Employers are responsible for conducting hazard assessments of all work tasks performed by employees to determine the necessary PPE for safe operations. When choosing the appropriate PPE for a task, many factors should be considered. These factors include the risk associated with the chemical, radiological materials, or biological agent/toxin (including concentration and quantity), any physical hazards that may be encountered, the routes of exposure, the material of the PPE, the permeation and degradation rates of a chemical, radiological materials or biological agent/toxin on the material, and the duration of time the PPE will be in contact with the hazard. Ensuring that PPE fits properly and comfortably is crucial for achieving optimal compliance and encouraging consistent use while performing a task. All PPE and clothing should be kept in good condition and should meet National Institute for Occupational Safety and Health (NIOSH) or American National Standards Institute (ANSI) standards, as applicable. The future of personal protective equipment (PPE) is ever evolving. Given the importance and benefit of PPE, there are several areas for improvement which may include: (1) Better design for comfort when required for long term or duration of usage, availability of different sizes for better fit, and breathability to manage heat stress when working in hot environment. (2) Enhanced training to ensure compliance, proper donning, and doffing, and understanding of limitations. (3) Better quality control and (4) Supply chain and availability or validated methods for reuse of PPE to support global crises (e.g., during a pandemic) and ensuring high quality and affordable product.
